# A systematic review of the effect of retention methods in population-based cohort studies

**DOI:** 10.1186/1471-2458-11-249

**Published:** 2011-04-19

**Authors:** Cara L Booker, Seeromanie Harding, Michaela Benzeval

**Affiliations:** 1 MRC/CSO Social and Public Health Sciences Unit, Glasgow, UK; 2 University of Essex, Institute for Social & Economic Research, Colchester, UK

## Abstract

**Background:**

Longitudinal studies are of aetiological and public health relevance but can be undermined by attrition. The aim of this paper was to identify effective retention strategies to increase participation in population-based cohort studies.

**Methods:**

Systematic review of the literature to identify prospective population-based cohort studies with health outcomes in which retention strategies had been evaluated.

**Results:**

Twenty-eight studies published up to January 2011 were included. Eleven of which were randomized controlled trials of retention strategies (RCT). Fifty-seven percent of the studies were postal, 21% in-person, 14% telephone and 7% had mixed data collection methods. A total of 45 different retention strategies were used, categorised as 1) incentives, 2) reminder methods, repeat visits or repeat questionnaires, alternative modes of data collection or 3) other methods. Incentives were associated with an increase in retention rates, which improved with greater incentive value. Whether cash was the most effective incentive was not clear from studies that compared cash and gifts of similar value. The average increase in retention rate was 12% for reminder letters, 5% for reminder calls and 12% for repeat questionnaires. Ten studies used alternative data collection methods, mainly as a last resort. All postal studies offered telephone interviews to non-responders, which increased retention rates by 3%. Studies that used face-to-face interviews increased their retention rates by 24% by offering alternative locations and modes of data collection.

**Conclusions:**

Incentives boosted retention rates in prospective cohort studies. Other methods appeared to have a beneficial effect but there was a general lack of a systematic approach to their evaluation.

## Background

Longitudinal cohort studies are important for the understanding of aetiological mechanisms underlying population and individual differences in the incidence of disease and for monitoring social inequalities in health[1,2]. Selective attrition, however, is a known problem in cohorts as those in disadvantaged socio-economic groups, ethnic minorities, younger and older people and those at greater risk of ill-health are more likely to drop out[3]. This may result the generalisability of findings being limited and estimates of association being biased[4]. Although direct evidence for this is limited with some studies[5] finding evidence of biased estimators with different response rates, while others found the associates were unaffected by selective attrition[6-8].

Overall, therefore, against a background of declining response rates in surveys in the UK[9] and in many cohort studies[10] more focused efforts to prevent attrition are required to ensure the benefit of the findings from cohort studies to public health do not become limited.

The barriers to recruitment and retention of participants in clinical trials are fairly well documented. General distrust of researchers and studies, concerns about research design, the consent process, discordance between lay beliefs and medical practice, patient treatment preferences, uncertainty about outcomes, and additional demands of the trial (e.g. duration of interventions, cost of travel, etc.) are frequently cited reasons for non-participation[11-13]. Similar reasons have recently been cited for non-participation in longitudinal cohort studies[14,15]. While a number of reviews have reported different ways of improving study participation, as well as the contextual factors that may affect these approaches,[16-18] little is known about the effectiveness of specific retention strategies, which may differ by study design. Reasons for attrition may differ for randomized trials, e.g. random assignment to an unwanted treatment group, and the process of randomization and the types of interventions, which, make it difficult to extrapolate the effectiveness of certain retention methods to cohort studies. Cohort studies are expensive, with the follow-up of high risk groups requiring the most effort and resources, so there is a critical need to identify effective retention strategies. For these reasons, in this review, we have decided to focus exclusively on cohort studies. The main objective of this review was to determine the effectiveness of retention strategies in improving retention rates in prospective population-based cohort studies.

## Methods

This review focused on the evaluation of retention strategies in prospective population-based cohort studies with health as an outcome. A population-based cohort was defined as "any well-defined population defined by geographic boundaries, membership or occupation"[19]. Studies were included if there was at least one wave of follow-up data collection in which the participant, or a proxy, was personally contacted by the study, at least one retention method was described and method-specific retention rates were reported. Studies were excluded if they were clinical or non-clinical trials evaluating the effectiveness of treatment regimens or intervention/prevention programmes, non-population-based cohort studies or cohorts with record linkage as the only method of follow-up. Only English language studies were searched and selected in order to reduce potential biases from misinterpretation. Studies which focus solely on locating (e.g. tracing) respondents, although an important activity for cohort maintenance, were not included in this review. Similar reviews of the effect of initial recruitment on subsequent retention have not been considered here, although again there is evidence that significant effort at recruitment may reduce subsequent attrition[20].

The electronic databases Medline, PsycINFO, PsycABSTRACTS, Embase, CINAHL, ISI, AMED and the Cochrane Central Register of Controlled Trials were initially searched for studies published through to June, 2007. The review was updated with additional searches of Medline, PsycINFO, ISI and the Cochrane Central Register of Controlled Trials conducted in November 2008 and in January 2011. Existing reviews were identified by searching the Cochrane library, the Database of Abstracts of Reviews of Effects (DARE) public databases at the Centre for Reviews and Dissemination, and other relevant medical and social science databases, including those produced by the Health Development Agency. Manual searches of bibliographies were also conducted to obtain reports on primary studies that were not retrieved through the electronic search. A list of potential prospective population-based cohort studies was also developed and study investigators were contacted and study websites searched for unpublished and technical reports.

Five terms were used in the electronic search 1) recruitment, 2) retention, 3) attrition, 4) participation, and 5) study design. In most cases a second keyword was included with the main search term, for example, attrition was paired up with any variation of "minimi" (e.g. minimization, minimizing, etc.). Use of the term 'recruitment' enabled identification of cohort studies that may have been missed through use of the retention term only. The search was restricted to include only publications where the two words were within two words of each other (i.e. minimization of attrition, attrition was minimized, etc.). Specific terms were agreed upon by the authors and adapted for each database (Additional File 1). CB conducted the initial appraisal of all titles and abstracts of papers. SH conducted a 20% re-check working independently to ensure that potentially relevant cohort studies or retention evaluations were not missed. Any disagreements were resolved through discussion. Data items to be extracted were agreed by all authors, and a data extraction database containing details of each study was developed by CB. MB independently reviewed all data extracted.

Retention strategies were categorised as 1) incentives to participate (monetary and non-monetary), 2) reminder calls or letters, repeat visits (i.e. more than one visit to schools to follow-up pupils who were not present on previous days of data collection) or repeat questionnaires and alternative modes of data collection and 3) other methods (e.g. method of posting, length of questionnaire). Differences in retention rates across the different retention strategies were examined with Meta-Analyst software[21]. Individual study proportions, that is the number of participants retained from a specific retention method divided by the number of participants approached, and 95% confidence intervals were calculated using random model analysis weighted by sample size and variance[21]. The individual study proportions given in the tables are the additional increase in the proportion of subjects retained from the specified method. Due to the heterogeneity of the methods within and between the RCTs and non-experimental studies meta-analyses were not conducted.

## Results

### Literature Search

**Figure 1:**
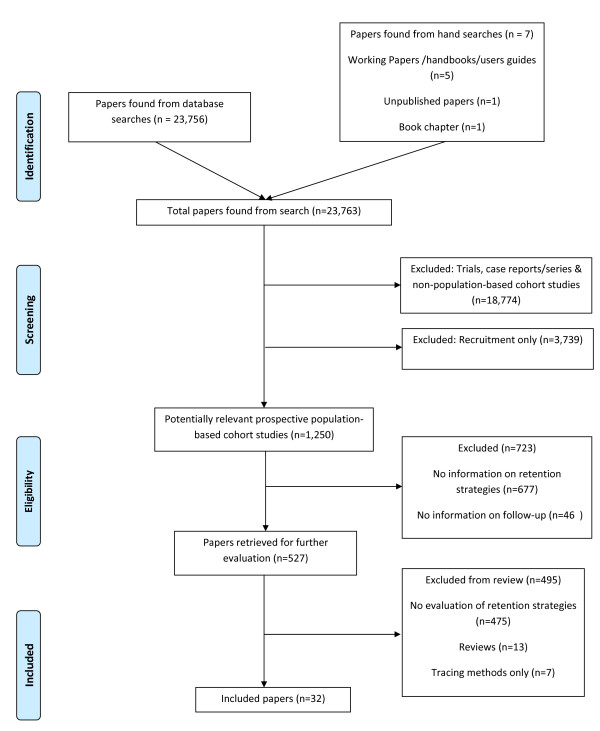
**Flowchart of search methodology**.

The literature, bibliography, and website searches, together with correspondence with study investigators identified 17 210 papers. As Figure 1 shows, the vast majority of these were excluded because they were not population-based studies or they focused on recruitment rather than retention strategies, leaving 913 potential papers. Two-thirds of these papers were excluded because they had no information on the retention strategies, 30% of which were then excluded as they did not contain information on the evaluation of retention strategies.

Twenty-eight studies from thirty-two papers, unpublished papers, technical reports, book chapters, and one personal communication were identified as eligible for inclusion in this review[22-53].,[Dudas, e-mail, 14 December 2007] Table 1 provides a description of each study (see Additional File 2 for a more detailed description).

**Table 1:** Description of Studies Included in Review

First Author (Publication Date), Study Name, Reference Number	Baseline Sample Population	Year Started	Baseline Sample Size	Interval Between Data Collections	Evaluation Year(s)	Data Collection Method	Retention Method (s)	**Response Rates**^**a,b**^
**Randomised Studies of Retention Strategies**								
Doody (2003) US Radiologic Technologists (USRT) Study, [22]	Adults	1984-1987*	132 454 2700 ^1^	8-10 Years	1995-1997	Postal	RCT of monetary incentives	72% 23% RCT
Kalsbeek (1995) 1993-1994 Self-Care Assessment of Community Based Elderly, [23]	Adults 67+	1991	3485	2 Years	1993-1994	Telephone	RCT of advance packets with non-monetary incentives	78%
Koo (1996) The Canadian Study of Diet, Life-Style and Health, [24]	Girls aged 5-13	1992	657	1 Year	1995	Postal	RCT of reminder letters	74% 55% RCT
Laurie (2007) British Household Panel Survey (BHPS), [25,26]	Households	1991*	10 264	1 Year	2004	Face-to-Face	RCT of monetary incentives	85%
Olson (2008) National Longitudinal Survey of Youth in 1979 (NLS79), [27-30]	Youth and young adults aged 14-22	1979*	12 686	1-2 Years	2000	Mixed	RCT of monetary incentives	83% 32% RCT
Olson (2008) National Longitudinal	Women aged 20-44	1967	5159	1-2 Years	2001	Mixed	RCT of monetary and non-monetary incentives	46%
Surveys of Young Women and Mature Women (NLSW), [27-30]	Women aged 14-24	1968	5083 766 ^1^	9 Years				54% 30% RCT
Olson (2008) National Longitudinal Survey of Youth in 1997 (NLS97), [27-30]	Youth aged 12-17	1997*	8984 3,825^1^	1 Year	2006	Face-to-Face	RCT of monetary incentives	84% 65% RCT
Rimm (1990) Health Professionals Follow-up Study, [31]	Males aged 40-75	1986*	51 672	1-2 Years	1988	Postal	RCT of mailing methods	93% 69% RCT
Rodgers (unpublished) Health and Retirement Study (HRS), [32]	Adults aged 60+	1992*	12 654	2 Years	2000	Telephone	RCT of monetary incentives	88%
White (2005) VITamins And Lifestyle (VITAL) Study, [33]	Adults aged 50-76	2000*	77 700 452 ^1^	2 Years	2002	Postal	RCT of non-monetary incentive	84% 34% RCT
**Non-Randomised Studies of Retention Strategies**								
Boys (2003), [34]	Youth aged 15-16	2000	540	1 Year	2001-2002	Postal	Reminder methods and alternative methods of data collection	92%
Calle (2002) Cancer Prevention Study II (CPS-II) Nutrition Cohort, [35]	Adults aged 50-74	1992*	184 194	2 Years	2003	Postal	Repeated questionnaire postings and alternative methods of data collection	90%
Clarke (1998) Whitehall I Study, [36]	Males aged 40-69	1967	19 019	25 Years	1995	Postal	Repeated questionnaire postings and reminder methods	73%
Eagan (2002), [37]	Adults aged 15-70	1985	3370	11 Years	1996-1997	Postal	Reminder methods	89%
Garcia (2005) Cornella Health Interview Survey Follow-up (CHIS.FU) Study, [38]	All ages	1994	2500	8 Years	2002	Telephone	Multiple telephone calls and alternative methods of data collection	68%
Harding (2007) The DASH (Determinants of Adolescent Social well-being and Health) Study, [39]	Youth aged 11-13	2003*	6643	2 Years	2005	Face-to-Face	Multiple school visits	72%
Hoffman (1998) CLUE II Study, [40]	Adults aged 18+	1989*	28411 2000 ^2 ^812 ^3^	6 Years	1995	Postal	RCT of questionnaire length, non-RCT of non-monetary incentives, non-RCT of re-posting of questionnaire and reminder postcard	72% 34% Pilot 1 46% Pilot 2 16% Pilot 3
Lissner (2003) Population Study of Women in Gothenburg, Sweden, [41]	Females aged 38-60	1968*	1462	6-8 Years	2000-2002	Face-to-Face	Alternative methods of data collection	72%
Michaud (2005) NIH-AARP Diet and Health Study, [42]	Adults aged 50-69	1995*	567 169	10 Years	2002	Postal	Multiple postings of questionnaire and alternative methods of data collection	76%
Mills (2000), [43]	Youth aged 11-12	1992	1614	1 Year	1995-1997	Face-to-Face	Alternative methods of data collection	91%
Novo (1999), [44]	Youth aged 16	1981*	1083	5 Years	1986	Face-to-Face	Alternative methods of data collection	98%
Rudy (1994), [45]	Women aged 20-35	1993	221	1 Month	1993	Postal	Non-RCT of monetary and non-monetary incentives	72%
Russell (2001) Black Women's Health Study (BWHS), [46]	Women aged 21-69	1995*	64 500	2 Years	1997	Postal	Multiple postings of questionnaire, reminder methods and alternative	83%
Tolusso (2003) National Population Health Survey (NPHS), [47]	All ages	1994*	17 276	2 Years	2003	Telephone	Multiple telephone calls and alternative methods of data collection	81%
Ullmann (1998) UCLA Study of Adolescent Growth, [48]	Youth aged 12-16	1976*	1634	1 Year	1992	Postal	Reminder methods, multiple postings of questionnaire and monetary incentive	68%
Walker (2000) British Regional Heart Study (BRHS), [49]	Males aged 40-59	1978*	7735	2-9 Years	1983-1985	Postal	Reminder methods	88%
Women's Health Australia Research Group (2001) Women's Health Australia (WHA) Study, [50-53]	Women aged 18-23 Women aged 45-50 Women aged 70-75	1996*	14 247 13 716 12 432	2 Years	1998-2000	Postal	Reminder methods and alternative methods of data collection	71% 92% 89%

^a ^Retention rates at time of publication

^b ^Overall study retention rates follow ed by retention rates from RCTs or pilot studies

° Personal communication

* Study still active

^1^Trial s amplesiz e

^2^Pilot 2

^3^Non-respondent to pilot 2

Of the 28 studies reviewed more than half were conducted in the USA, were postal questionnaires and were conducted with adult cohorts. The majority were less than 10 years old when the retention strategy took place and had less than 10 follow ups.

Of the 28 studies, 11 were RCTs,[22-33] of which 9 focused on the effectiveness of incentives and 2 experimented with the interview length and postal methods; the remaining 17 conducted other types of analyses of the effectiveness of the retention methods used[34-53].,[Dudas, e-mail, 14 December 2007] Some of the retention efforts were conducted in pilot studies[36,40,42] and others were trialled after the main attempt data collection had been completed[22,24,27-31,33]. This use of retention methods on sub-populations may have a significant effect on the response rates reported. For example, pilots on hard-to-reach groups or reluctant participants may have low response rates; however, the addition of these participants could improve both the overall response rate and representativeness of the study population.

### Incentives

Incentives were evaluated in ten studies,[22,23,25-28,32,33,40,45] the results of which are shown in Table 2. Incentives were associated with an increase in overall retention rates[22,25-28,32,33,45]. Five studies[23,25,26,32,40,45] trialled incentives with all study participants, i.e. incentives were included in the first data collection attempt. The increases associated with the provision of, or increase in the value of incentives, ranged from 2% to 13%. Two studies[25,26,32] randomized differing amounts of monetary incentives and results showed that retention was higher in groups that received higher amounts. Two studies[23,40] examined the effects of non-monetary incentives on retention and found no increase in retention. However, Rudy et al. in a non-experimental study, reported a 79% response rate for those receiving $100 incentive and 66% for those receiving non-cash gifts (*X*^2 ^(1,166) p < 0.05)[45]. Retention rates also increased with the value of monetary incentive offered[22,25-28,32,33].

**Table 2:** Increase in Study Retention Rates for Incentive and Reminder Letters by Data Collection Type

		Data Collection Method		
	
	Postal	Face-to-Face	Telephone	Mixed
	
**Evaluated Retention Method**, ^**reference number**^	Average increase in retention rate, proportion (95% CI)	Average increase in retention rate, proportion (95% CI)	Average increase in retention rate, proportion (95% CI)	Average increase in retention rate, proportion (95% CI)
**Incentives**				
*RCT - Financial Only*				
Doody[22]*	0.01 (0.01, 0.01)			
Olsen (NLS79)[27-30]*				0.05 (0.05, 0.06)
Olsen (NLSW)[27-30]*				0.02 (0.02, 0.03)
Laurie[25,26]***		0.85 (0.84, 0.85)		
Rodgers[32]***			0.80 (0.87, 0.88)	
*RCT - Gift Only*				
Kalsbeek[23]***			0.78 (0.76, 0.79)	
White[33]*	0.11 (0.01, 0.14)			
*RCT - Mixed*				
Olsen (NLS97)[27-30]*		0.28 (0.27, 0.29)		
*Non-RCT - Gift Only*				
Hoffman[40]	0.47 (0.44, 0.49)			
*Non-RCT - Mixed*				
Rudy [45]***	0.72 (0.65, 0.79)			
**Reminder Letters**				
*1 Letter Posted*				
Boys[34]	0.12 (0.10, 0.15)			
Hoffman[40]	0.02 (0.02, 0.03)			
Koo[24]**	0.32 (0.29, 0.36)			
Russell[46]	0.03 (0.03, 0.03)			
*2 Letters Posted*				
Clarke[36]	0.18 (0.15, 0.22)			
Eagan[37]	0.18 (0.17, 0.19)			
Walker[49]	0.18 (0.17, 0.19)			
WHA Research Group (YC)[50-53]	0.03 (0.03, 0.03)			
WHA Research Group (MC)[50-53]	0.10 (0.10, 0.11)			
WHA Research Group (OC)[50-53]	0.46 (0.45, 0.47)			
*3 Letters Posted*				
Ullman[48]	0.23 (0.20, 0.26)			

* Increase in overall retention with the addition of respondents from the RCT with initial non-responders

** RCT of reminder letters

*** Retention rate of entire sample

An exception to the finding of increased response with greater monetary value was the study by Doody et al. which, found that a $2 bill resulted in a higher retention rate than a $5 cheque[22]. One possible explanation is that in the United States, a $2 bill is rare and may have novelty; alternatively, the higher amount was given as a cheque and the transaction cost of cashing a low-value cheque may have reduced the effect of the incentive[22]. The National Longitudinal Surveys (NLS) found that not all respondents who received cheques or cash cards as incentives used them. Although equivalent cash value were not offered to provide a direct comparison, it is likely that while reducing the overall cost of incentives to the study,[27,28] this strategy also reduced the impact of an incentive on the retention rate. There is tentative evidence that providing incentives, particularly upfront to specific groups, e.g. non-responders to previous data collection, may reduce the cost per interview as the cost of the incentive is cheaper than multiple visits or calls to obtain such respondents without an incentive[27,28].

### Reminders, repeat contacts and alternative modes of data collection

The most common approach to improving retention was to write to or call respondents to remind them to complete a questionnaire or take part in an interview; to send additional questionnaires, make repeat calls or visits; or, to offer alternative modes of data collection in an attempt to capture reluctant respondents. Seventeen studies included at least one of these methods,[22,24,33-44,46-53] most including a range of them in a hierarchical fashion, starting with the least labour intensive (i.e. reminders) and ending with the most costly (i.e. alternative modes of data collection). With the exception of one study[42] it was possible to separate out the effect of each specific stage on retention.

Table 2 shows the additional proportion retained after posting reminder letters or postcard following a postal survey, which appeared to increase with number of letters sent. The time between sending out the initial postal questionnaire and the reminder varied by study, but no study evaluated the optimal time between postings.

Ten studies posted questionnaires to participants multiple times,[22,31,33,35-37,40,42,46,48], [Dudas, e-mail, 14 December 2007] with nine[22,31,33,35-37,40,46,48], [Dudas, e-mail, 14 December 2007] providing retention rates for each posting. Table 3 shows that the additional proportion retained from posting repeat questionnaires appeared to increase with the number posted. Only one study compared the effectiveness of reminder letters with that of repeat questionnaires. Hoffman et al. found that those who received a second questionnaire were much more likely to be retained than those who received only a reminder postcard[40].

**Table 3:** Increase in Study Retention Rates for Repeat Questionnaires and Alternative Methods of Data Collection by Data Collection Type

		Data Collection Method		
	
	Postal	Face-to-Face	Telephone	Mixed
	
**Evaluated Retention Method**, ^**reference number**^	Average increase in retention rate, proportion (95% CI)	Average increase in retention rate, proportion (95% CI)	Average increase in retention rate, proportion (95% CI)	Average increase in retention rate, proportion (95% CI)
**Repeat Questionnaires**				
*2 Questionniares Posted*				
Doody[22]*	0.08 (0.08, 0.08)			
Eagan[37]	0.18 (0.17, 0.19)			
Hoffman[40]	0.05 (0.04, 0.06)			
Rimm[31]	0.16 (0.16, 0.17)			
Ullman[48]	0.04 (0.03, 0.06)			
*3 Questionniares Posted*				
Clarke[36]	0.18 (0.15, 0.22)			
White[33]*	0.06 (0.04, 0.08)			
*6 Questionnaires Posted*				
Calle[35]	0.37 (0.37, 0.38)			
Russell[46]	0.23 (0.23, 0.23)			
**Alternative Methods of Data Collection**				
*Postal Questionnaires*				
Garcia[38]			0.01 (0.01, 0.02)	
Mills[43]		0.17 (0.15, 0.19)		
*Face-to-Face Interviews*				
Lissner[41]		0.18 (0.16, 0.21)		
Tolus so[47]			0.02 (0.02, 0.02)	
*Telephone Interviews*				
Boys[34]	0.01 (0.01, 0.03)			
Calle[35]	0.02 (0.02, 0.02)			
Michaud[42]	0.17 (0.16, 0.18)			
Russell[46]	0.02 (0.02, 0.02)			
WHA Research Group (YC)[50-53]	0.01 (0.01, 0.01)			
WHA Research Group (MC)[50-53]	0.05 (0.05, 0.05)			
WHA Research Group (OC)[50-53]	0.08 (0.08, 0.09)			
*Mixed (Postal, Telephone & Face-to-Face) *Novo[44]		0.42 (0.39, 0.44)		

Ten[34,35,38,41-44,46,47,50-53] of the twenty-eight studies offered alternative data collection modes to participants; seven[34,35,38,42,46,47,50-53] of these studies had already used other retention methods. Table 3 shows that there was an increase in retention with any alternative additional data collection method. The additional retention was highest for face-to-face studies,[41,43,44] which conducted the first interview in a central location e.g. a clinic or school and the subsequent modes either followed up with home interviews or postal questionnaires[41,43,44]. In these studies, alternative modes of data collection were generally the second stage of the study, which in addition to the convenience of home-based interviews might help to explain the larger average increases in retention[41,43,44].

The increase in retention from reminder calls made for postal survey studies was also examined in four studies (data not shown); all of which had already sent reminder letters[34,37,46,50-53]. Reminder calls appeared to have a greater effect on younger age cohorts,[34,50-53] with an increase of between 10% and 16%, in comparison to increases between 1% and 6% among older cohorts[37,46,50-53] retention only.

Two telephone surveys demonstrated the need to make multiple calls to achieve a completed interview (data not shown)[38,47]. Garcia and colleagues found 62% of participants only required between one and three telephone calls to complete an interview. However, ten or more calls, however, were required to successfully interview 9% of participants[38]. The National Population Health Survey found that less than 15 attempts were needed to conduct 90% of the interviews; however, up to 50 calls were required for the remaining 10%[47].

In a school-based study of multi-ethnic pupils in England, Harding et al., (data not shown) used multiple school visits (i.e. up to 13 additional visits to schools to follow-up pupils who were not present on previous data collections). Retention increased by 26%, 6% and 2% after the second, third and fourth visits respectively[39].

### Multiple Methods

Thirteen postal survey studies[22,24,33-37,40,42,46,48-53] in this review used multiple retention methods and eleven of these[22,24,33-37,42,46,49-53] had retention rates of more than 70%. This might suggest that the more effort studies put into retaining respondents, including use of multiple methods, the higher the retention rate will be. However, it is important to keep in mind the costs will also be higher. These studies[22,24,33-37,40,42,46,48-53] all began with the cheapest methods, e.g. posting reminder letters, and ended with the most expensive, such as alternative modes of data collection, so that the number of respondents that needed to be captured with each additional method decreased as the costs increased.

### Other methods

In addition to the broad approaches described above a few more specific initiatives were tried by some studies. Rimm et al. found that retention was significantly higher for questionnaires sent via certified mail than those sent by other mail types, and for envelopes that were handwritten than those that were not[31]. However, Doody et al. did not find any difference in retention from alternative methods of posting the questionnaire[22]. Kalsbeek and Joens reported some evidence of increased retention by providing personalised information in letters, however as this was combined with non-monetary incentives, determining the true effect of personalization was difficult[23].

In two more detailed experiments, Hoffman et al. found a modest increase in retention if a 4-page questionnaire was used instead of a 16-page questionnaire (p = 0.145);[40] Clarke et al. found that including income questions did not affect retention rates but asking for proxy respondents to complete a cognitive questionnaire about the primary study participants appeared to decreased retention when the main questionnaire was used[36].

## Discussion

In the studies reviewed here, incentives were associated with an increase in retention rates, which improved with higher values. Whether cash was the most effective incentive was not clear from studies that compared cash and gifts of similar value. Studies of other methods (i.e. reminder letters or calls and alternative modes of data collection) also demonstrated a benefit, but it was difficult to assess their impact due to a less standardised approach. This is the first-known review of the effect of retention strategies on retention rates specifically focused on population-based cohort studies. It is important to consider the effect of different retention strategies on longitudinal studies specifically as different mechanisms may operate once a participant has been/or expects to be in a study over a period of time.

### Strengths and Limitations

A major strength of this review is its extensive systematic search of the literature. The general lack of studies that rigorously evaluate retention methods suggests, therefore, that such evaluations are rarely conducted. A key challenge in this review has been comparing retention rates from studies with different methods of calculating or reporting them. In general, we used the retention rate as reported by the authors but we are aware that different methods of calculation could have been used. For example, among studies with two or more follow-up data collections, some used the number of participants eligible for a specific wave of data collection as the denominator[41,48] while others used the baseline sample[34,43,44,49]. An additional difficulty was created by the inclusion of new or additional participants between waves (e.g. "booster samples", new members in the household, previous non-responders or drop-outs, or studies adopting a policy of continuous recruitment) and whether they were included or excluded from the denominator.

The majority of studies in this review were conducted in the United States which may limit their generalisability to studies conducted in other countries, which, may have different cultures about participating in research or have different ethical guidelines. We also attempted to examine whether the time between data collections or between baseline data collection and the evaluation wave may have influenced retention. However, due to the small number of studies involved and heterogeneity between them, the findings were not reliable.

Compositional factors such as the gender, age and socioeconomic status of participants and contextual factors such as location of the study, the recruitment methods the tracking methods or other indirect methods such as study loyalty or study publicity may also have influenced the effect of retention methods. Few studies had empirical data or reported these in a systematic way so that evaluation or aggregation could be conducted across studies. However, there is some evidence that additional efforts are required to track and retain vulnerable [54] and/or disadvantaged groups[5]. The differential effect of different retention methods across population groups therefore requires further systematic review.

The use of a narrative approach for this review rather than meta-analysis was due to the relatively small number of studies and the heterogeneity in their methodologies, which limited our ability to quantify effects associated with specific retention methods and carry-out meta-analyses. The findings of this study demonstrate the need for studies to rigorously evaluate their retention methods as well as examine the cost-effectiveness of those methods.

### Comparisons of effects of similar methods used in non-cohort studies

In other research, incentives have been shown to have a positive effect on retention rates in postal surveys,[55-57] other study designs, in-person and telephone interviews and online studies,[58-60] and also in the recruitment of study members in longitudinal cohort and cross-sectional studies and clinical trials[61-64]. In a meta-analysis of the methods used in postal studies to increase response and retention rates, the odds of response or retention increased by more than half when monetary incentives were received versus non-monetary incentives[57]. Our review was inconclusive in relation to cash versus gift incentives but it did suggest that cash incentives may have a greater effect than cheques on retention rates. There is some support for this in other studies,[65-71] although they are from health promotion and health care projects rather than epidemiological research studies, and therefore their findings may not be transferable to research studies.

The effect of the timing of incentives on retention rates in our review was unclear. However, the meta-analysis by Edwards et al. showed that prepaid incentives increased response more than conditional incentives (OR = 1.61 [1.36,1.89]) in studies that posted questionnaires[57]. Unconditional incentives were also found to lower attrition in a postal questionnaires[70]. In a recent review of studies that used either face-to-face or telephone interviews, conditional incentives did not increase response compared to unconditional (β = 2.82, SE = 1.78, p > 0.05)[59].

Although there was a general lack of standardised approaches of evaluating other retention methods here, there is support in the literature for a beneficial effect on response and retention rates of reminder methods in trials, cross-sectional, prospective and non-population based cohort studies[57,72-80].

A recent systematic review of retention methods used for in-person follow-up showed that retention rates increased with the number of methods used,[81] which is supported by our findings. The relative lack of evaluation of retention strategies in cohort studies is possibly linked to funding constraints as well as to the potential threat of compromised retention from employing control arms. The use of sub-studies[22,27-30] or pilot studies[36,40,42] provided useful insights about how retention can be enhanced by the evaluation of methods without compromising retention rates. Olson argued that targeted strategies, such as incentives to non-responders from previous waves of the study, is a cost effective approach to retaining those participants who often drop out of studies[27,28].

## Conclusions

Producing generalisable results is a key objective of cohort studies to ensure that the benefits of research can be applied to a wider population. Researchers are encouraged to ensure that participants are given the opportunity to take part and are not excluded due to socioeconomic disadvantage. Much has been written on the ethics of incentives,[82-84] but there is still a lack of consensus, for example, whether varying incentives amounts should be offered to different sub-samples in a study. There is little ethical discussion about whether repeated attempts to obtain consent to follow-up is perceived as pressure to participate or whether research ethics should be adapted to suit the cultural/socio-economic characteristics of the study population. Due to international differences in the regulation of research, the approach to these issues will invariably vary. There was little mention of these issues in the studies we reviewed.

The cost of evaluation, and the risk to study loyalty among participants, may explain the small number of studies that evaluated retention strategies or examined their cost-effectiveness. Raising awareness of the need for such studies among researchers and funding bodies is important to ensure the longevity and scientific value of cohort studies in the future.

## Competing interests

The authors declare that they have no competing interests.

## Authors' contributions

CB was involved in the development of the concept for the paper, oversaw the literature search and conducted the data extraction. She also conducted the analyses, prepared drafts and undertook edits. SH was involved in the development of the concept, direction, drafting and editing of the manuscript. She also rechecked the abstracts for study inclusion. MB was involved in the direction and concept of the paper. She independently conducted data extraction and was involved in the drafting and editing of manuscript drafts. All authors have read and approved of all versions of the manuscript.

## Pre-publication history

The pre-publication history for this paper can be accessed here:

http://www.biomedcentral.com/1471-2458/11/249/prepub

## Supplementary Material

Additional file 1 **Is an example of the electronic database search for retention/attrition in cohort studies**. Click here for file

Additional file 2 **Is an extended version of Table **1**with additional information on the evaluation method and retention rates associated with those methods**. Click here for file
