# Supplementary material for: A systematic review of the effect of retention methods in population-based cohort studies
Source: BMC Public Health. 2011 Apr 19;11:249. doi: 10.1186/1471-2458-11-249 (PMC3103452; doi:10.1186/1471-2458-11-249)
Supplement: Additional file 1 — Is an example of the electronic database search for retention/attrition in cohort studies. [file 1471-2458-11-249-S1.DOC]

**Additional File 1.** Sample search for cohort studies.

**[Attrition terms**

i (minimi$ adj2 attrition) or (prevent$ adj2 attrition) or (lessen$ adj2 attrition) or (decreas$ adj2 attrition) or (reduc$ adj2 attrition)

OR

ii (loss or lost) adj2 (follow-up or followup)

OR

**Retention terms**

iv (increas$ or encourag$ or maximi$ or promot$) adj2 retention

OR

v (retention adj2 strateg$) or retention rate or (difficult$ adj2 (retain$ or retention))]

AND

**Study-type terms**

vi longitudinal or follow-up stud$ or followup stud$ or cohort$

Limit to English language

Notes:

1. patient dropouts/ is a MeSH heading - only applicable to Medline.

In Embase it maps to the term Patient which is too broad so was not used.

For CINAHL, Research subject retention/ used since 1994

For PSYCINF0, Experimental attrition/ used since 1995

2. ADJ2 - means that the terms are picked up when they occur within 2 words of each other, in either direction eg difficult to retain; strategy for retention, reducing levels of attrition. ADJ2 used in Medline, ADJ3 used in all other Ovid databases.

WITHIN 3 was used in the CSA databases

In WoK, the operator SAME is used which identifies terms in the same sentence.
