# Supplementary material for: A systematic review of the effect of retention methods in population-based cohort studies
Source: BMC Public Health. 2011 Apr 19;11:249. doi: 10.1186/1471-2458-11-249 (PMC3103452; doi:10.1186/1471-2458-11-249)
Supplement: Supplementary file 2 [file 1471-2458-11-249-S2.DOC]

Additional Table 1. Detailed description of studies included in review

First Author (Publication Date), Study Name, Reference Number	Study Description	Evaluated Retention Strategy	Outcome of Retention Strategy Evaluation,[a,b]
		**Randomised Studies of Retention Strategies**	
Doody (2003)<br>US Radiologic Technologists (USRT) Study, [22]	132 454 Certified radiologic technologists (adults) in the United States<br>Started in 1984-1987<br>Postal questionnaires on cancer risk<br><br>Evaluation Wave: First follow-up (1995-1997) on 126 628 eligible baseline participants<br>Following two postings of the first follow-up survey and telephone reminders to 31% of nonresponders, a randomised control trial (RCT) of incentives and postal method conducted with all remaining nonresponders	1. 2700 randomly selected non-responders randomised to monetary incentives of $0, $1.00 cash, $2.00 cash or cheque and $5.00 cheque<br><br>2. Incentives were posted via randomised mail delivery method of US first-class mail or Federal Express<br><br>Following RCT, $1 bill was sent by first-class to remaining nonresponders, 45 576, following an advance letter	17% response rate for those who did not receive any incentive, 25% for the $1 bill, 22% for the $2 cheque, 29% for $2 bill and 28% for $5 cheque, across both mailing methods<br><br>23% RCT response rate<br><br>$1 bill sent to remaining nonresponders increased overall retention rate by 8%<br><br>72% overall retention rate
Kalsbeek (1995)<br>1993-1994 Self-Care Assessment of Community Based Elderly, [23]	3485 non-institutionalised elderly (67+ years) in the United States<br>Started in 1991<br>Computer-assisted telephone interviews and proxy interviews on self-care behaviours<br>Evaluation Wave: First follow-up (1993-1994) on 3172 eligible participants<br>RCT of advance packets (i.e. letters and flyers sent prior to telephone interview)	1. Randomised participants to receive one of three types of advance packets (i.e. letters and flyers) with varied levels of personalization and a non-monetary incentive<br><br>Packet were either personalised with magnet; personalised, without magnet; and non-personalised, without magnet	81% retention rate for those who received the personalised packet without magnet, 76% for those who receive the personalised packet with magnet, and 77% for those who received the nonpersonalised packet, without magnet<br><br>Agreement rate (i.e. completed interviews and cognitively impaired ineligible consenters) did not differ significantly across advance packet groups<br><br>78% overall retention rate
Koo (1996)<br>The Canadian Study of Diet, Life-Style and Health, [24]	657 females (5-13 years) in Canada<br>Started in 1992<br>Postal questionnaires and phone interviews on dietary and lifestyle determinants of the onset of menarche<br><br>Evaluation Wave: Third annual follow-up (1995) of 615 eligible participants<br>After first posting of questionnaire conducted an RCT of reminder letters with advance notification of phone contact to parents of non-responders	1. Randomised three types of reminder letters included with second mailing of questionnaire (posted 22 days after initial post) to 358 non-responders<br><br>Reminder letters were either regular reminder letter (no additional reminders); reminder letter with an additional phone reminder indicated; reminder letter with an additional phone interview indicated	62% response rate for those who received reminder letter with an additional phone reminder indicated, 53% for those who received the reminder letter and phone interview indicated, and 52% those who received the reminder letter only<br><br>55% RCT response rate<br><br>74% overall retention rate

Laurie (2007)<br>British Household Panel Survey (BHPS), [25,26]	10 264 people (16+ years) in 5505 households in the UK<br>Started in 1991<br>In-person interviews on housing, mobility, education, labour force participation and health<br><br>Evaluation Wave: Thirteenth follow-up (2004) of eligible participants<br>RCT of value of monetary incentive	1. Randomised monetary incentives of £10 or £7 gift voucher for adults (increase of incentive amount from previously offered amount was £7)	92% of previous round responders who were offered £7 completed an interview compared to 94% who received £10<br><br>6% of previous round non-responders who were offered £7 completed an interview compared to 13% who received £10<br><br>Within household refusal rates was lower for the £10 group, 3%, compared to the £7 at 10%<br><br>85% overall retention rate
Olson (2008)<br>National Longitudinal Survey of Youth in 1979 (NLS79), [27-30]	12 686 representative young people (14-22 years) in the United States<br>Started in 1979<br>Telephone interview, or in-person interview or web survey on youth unemployment, and transition from school to work and labour force participation<br><br>Evaluation Wave: Nineteenth follow-up (2000) of eligible participants<br>RCT of monetary and non-monetary incentives to uncompleted participants	1. Randomised monetary and non-monetary incentives of $40 cash or $80 cash to 2086 reluctant participants (increase of incentive amount from previously offered $20)	8% of previous round non-respondents who were offered $40 completed an interview compared to 43% for previous respondents<br><br>14% of previous round non-respondents who were offered $80 completed an interview compared to 50% for previous respondents<br><br>32% RCT response rate<br><br>83% overall retention rate
Olson (2008)<br>National Longitudinal Surveys of Young Women and Mature Women (NLSW), [27-30]	5159 mature females (20-44 years) re-entering the workforce and 5083 young females (14-24 years) entering the work force for the first time in the United States<br>Started in 1967<br>Telephone and in-person interviews on balancing roles, labour force participation and fertility<br><br>Evaluation Wave: Twentieth (mature women) and twenty-first (young women) follow-ups (2001) of<br>Pilot (i.e. conducted prior to full data collection) RCT of monetary and non-monetary incentives on 1099 previous round non-responders	1. 766 randomised selected participants randomised to monetary incentives of $0, $20, or $40 cash cards	38% of women who received $40 completed an interview compared to 31% who received $20 and 19% who were not offered an incentive<br><br>30% RCT response rate<br><br>46% overall retention rate for mature females<br><br>54% overall retention rate for young females

Olson (2008)<br>National Longitudinal Survey of Youth in 1997 (NLS97), [27-30]	8984 representative young people (12-17 years) in the United States<br>Started in 1997<br>Computer-assisted personal interview on youth unemployment, transition from school to work and labour force participation<br><br>Evaluation Wave: Tenth follow-up (2006) of eligible participants<br>RCT of monetary and non-monetary incentives on difficult, uncompleted cases	1. 3825 previous round participants &and non-participants randomised to incentives of $10 cash, $30 cash, or $30 in gifts	Of the respondents to the previous round, 86% responded who received $30 cash compared to 85% who received $30 in gifts, and 82% who received $10 cash<br><br>Of the non-respondents to the previous round, 35% responded who received $30 cash compared to 24% who received $30 in gifts, and 28% who received $10 cash<br><br>65% RCT response rate<br><br>84% overall retention rate
Rimm (1990)<br>Health Professionals Follow-up Study, [31]	51 672 male health professionals (40-75 years) in the United States<br>Started in 1986<br>Postal questionnaires on diet and heart disease and cancer risk factors<br>69% overall response rate<br><br>Evaluation Wave: First follow-up (1988) of baseline participants<br>After three postings of questionnaire, conducted an RCT on the 12 233 non-respondents	Phase 1: Randomised 1195 randomly selected non-responders (10% of non-responders) of first follow-up to postal methods of certified mail, United Parcel Service (UPS), window envelopes with personal return address, typewritten addressed envelopes, and hand-addressed envelopes; remaining 11 038 non-responders (90% of non-responders) assigned to standard window envelope (control group)<br><br>2. Phase 2: Following RCT, questionnaire were sent via certified mail to remaining 8889 non-responders	63% response rate for certified mail; 47% for UPS, 24% for window envelope, 28% for typed address, 42% for handwritten envelopes, and 26% for standard window envelope<br><br>27% response rate for phase 1<br><br>58% response rate for phase 2<br><br>69% response rate to phases 1 and 2<br><br>Increased retention rate by 17% from first three postings with addition of RCT responders<br><br>93% overall retention rate
Rodgers (unpublished)<br>Health and Retirement Study (HRS), [32]	12 654 adults (60+ years) in the United States<br>Started in 1992<br>Telephone interviews on housing, labour force participation, retirement, cognition, family and health<br><br>Evaluation Wave: Fourth follow-up (2000)<br>RCT of monetary incentives divided participants into four strata: deteriorating health; proxy informant at previous wave; non-responder at previous wave; all other eligible participants	1. Randomised eligible participants to $20, $30 or $50 (increase of incentive amount from previously offered $20)	Among those with deteriorating health, retention was 93% for $20, 95% for $30 and 97% for $50 (significant difference between $50 and $20 retention rates)<br><br>Among those with proxy informant at previous wave, retention was 92% for $20, 89% for $30 and 94% for $50 (significant difference between $50 and $30 retention rates)<br><br>Among non-responders at previous wave, retention was 38% for $20, 38% for $30 and 45% for $50 (significant difference between $50 and $20 retention rates)<br><br>Among all other eligible participants, retention was 94% for $20, 98% for $30 and 98% for $50 (significant difference between $50 and $20 retention rates)<br><br>88% overall retention rate

White (2005)<br>VITamins And Lifestyle (VITAL) Study, [33]	77 700 adults (50-76 years) in the United States<br>Started in 2000<br>Postal questionnaire on association of vitamin supplement use with cancer risk<br><br>Evaluation Wave: First follow-up (2002) pilot response study of 439 eligible respondents<br>After one posting of questionnaire, personalised letter and self-addressed envelope and a reminder postcard 2 weeks later, conducted an RCT of non-monetary incentive on pilot non-respondents	1. Randomised 141 non-responders of the pilot to second posting of questionnaire and non-monetary incentive of study-logo pencil or no pencil<br><br>Third posting of questionnaire followed 2 months after RCT	Of non-responders, participants who received a pencil were significantly more likely to complete a questionnaire, 44% compared to 24% of those who received no pencil<br><br>34% RCT response rate<br><br>68% of pilot participants completed a questionnaire prior to RCT<br><br>Cumulative response for those who received two questionnaires and pencil was 82% compared to 76% for those who received only two questionnaires<br><br>26% response from third posting of questionnaire<br><br>84% overall retention rate
	**Non-Randomised Studies of Retention Strategies**		
Boys (2003), [34]	540 year 11 students (15-16 years) in England<br>Started in 2000<br>Postal questionnaires and telephone interviews on adolescent drinking<br>96% response at first follow-up and 92% at second<br><br>Evaluation Waves: First follow-up (2001) and second follow-up (2002) of baseline participants<br>Offered entry into prize draw and £10 gift vouchers for completed questionnaires (not evaluated)	1. Reminder postcards sent two weeks after questionnaire posting<br><br>2. Reminder telephone calls made one week after posting of reminder postcards<br><br>3. Alternative methods of data collection of telephone interviews, for non-responders to the reminder postcards and calls, offered six weeks after posting of questionnaire	Reminder postcards increased retention rate by an additional 17% at the first follow-up and 12% for the second follow-up<br><br>Reminder telephone calls increased retention by an additional 7% in the first follow-up and 10% in the second<br><br>Telephone interviews increased retention only by 6% in the first follow-up and 1% in the second<br><br>96% overall retention rate at first follow-up and 92% at second
Calle (2002)<br>Cancer Prevention Study II (CPS-II) Nutrition Cohort, [35,°]	184 194 adults (50-74 years) in the United States<br>Started in 1992<br>Postal and phone questionnaires on diet, lifestyle factors, cancer incidence and mortality<br><br>Evaluation Wave: Fifth follow-up (2003) of all eligible participants	1. Up to 6 postings of questionnaires<br><br>First four postings are long form questionnaires, fifth and sixth postings are short form and sixth posting is sent via certified mail<br><br>2. Alternative methods of data collection of telephone interviews	On average, 65% completed questionnaire after first mailing, retention increased by an additional 10%, 4%, 2%, 2%, and 5% for the second through sixth mailings, respectively<br><br>Administering a short questionnaire by phone increased retention by 2%<br><br>90% overall retention rate

Clarke (1998)<br>Whitehall I Study, [36]	19 019 male adults (aged 40-69 years) in England<br>Started in 1967<br>Postal questionnaires on health behaviours and practices<br><br>Evaluation Wave: First full re-survey (1995) pilot of 401 randomly selected eligible participants	1.Second posting of questionnaire and reminder letter sent one month after first posting<br><br>2. Second posting of reminder letters, posted “recorded” delivery	55% returned questionnaires without reminders (from first posting of questionnaire)<br><br>12% returned after second posting of questionnaire and first reminder letter<br><br>6% additional retained after second reminder letter<br><br>73% overall retention rate
Eagan (2002), [37]	3370 randomly selected adults (15-70 years) in Norway<br>Started in 1985<br>Postal questionnaires on respiratory health<br><br>Evaluation Wave: First follow-up (1996-1997) of 3181 eligible participants<br>Postal questionnaire with invitation to clinical examination	1. Central Population Register<br><br>2. First reminder letter and second questionnaire sent if participant did not attend scheduled clinical exam<br><br>3. Second reminder letter and third questionnaire sent to non-responders of first reminder<br><br>4. Reminder telephone calls by physicians to non-responders of second reminder	Central Population Register did not find <1% of participants<br><br>65% returned questionnaire without reminder letters<br><br>14% returned after receiving first reminder letter<br><br>4% returned after second reminder letter<br><br>5% additional retained after telephone reminder<br><br>89% overall retention rate
Garcia (2005)<br>Cornella Health Interview Survey Follow-up (CHIS.FU) Study, [38]	2500 randomly selected non-institutionalised persons (all ages) in Spain<br>Started in 1994<br>Telephone interviews and postal questionnaires on smoking habits, alcohol consumption and physical activity level<br><br>Evaluation Wave: First follow-up (2002) of 2150 eligible participants	1. Local census<br><br>2. Multiple (up to 15) telephone calls<br><br>3. Alternative methods of data collection of postal questionnaire to un-reached participants	Local census found 100% of participants<br><br>Participants who were traced required 4.0 phone calls compared to 8.4 calls to non-traced participants<br><br>Most participants needed 1-3 calls, however, 9% needed 10+ calls to be traced<br><br>67% retention to telephone interviews<br><br>1% additional retained from postal questionnaire<br><br>68% overall retention rate
Harding (2007)<br>The DASH (Determinants of Adolescent Social well-being and Health) Study, [39]	6643 multiethnic students (11-13 years) in England<br>Started in 2003<br>School questionnaires on health and social conditions associated with ethnic differences and chronic disease in later life<br><br>Evaluation Wave: First follow-up (2005) of 6205	1. Multiple visits (up to 13) to schools	52% completed questionnaires in the first school visit, 36% in the second, 9% in the third, and 3% were completed in four or more school visits<br><br>72% overall retention rate

Hoffman (1998)<br>CLUE II Study, [40]	28 411 adults (18+) in the United States<br>Started in 1989<br>Postal questionnaire on risk factors of cancer and stroke<br><br>Evaluation Wave: Three pilots for first follow-up (1995) eligible participants	1. Pilot 1: 2174 baseline participants living outside of the active follow-up area (48.3 km) assigned to receive full, 16 pages, or short, 4 pages, of questionnaire<br><br>2. Pilot 2: 2000 pilot participants assigned non-monetary incentives of research unit pencil, copy of newspaper article on study, both article and pencil, and no incentive<br><br>3. Pilot 3: 812 non-responders to pilot 2, half assigned to receive a second posting of the questionnaire along with a reminder letter, posted seven weeks after first questionnaire posting and one have received a reminder postcard (9 weeks after initial posting)	Retention rate for the short questionnaire was 37% compared to 34% for the full version<br><br>34% retention rate for pilot 1<br><br>45% retention rate for no incentives, 46% for newspaper article, 47% for pencil and 48% for both<br><br>46% retention rate to pilot 2<br><br>Response was significantly higher among those who received a second questionnaire, 23% compared to those who received a reminder postcard, 10%<br><br>16% response rate to pilot 3<br><br>53% overall retention to pilots 2 and 3
Lissner (2003)<br>Population Study of Women in Gothenburg, Sweden, [41]	1462 females (38-60 years) in Sweden<br>Started in 1968<br>Clinic and home visits on health<br><br>Evaluation Wave: Fourth follow-up (2000-2002) of 929 eligible participants	1. Alternative methods of data collection of home visit examinations	Home visit examinations increased retention rate by 18%<br><br>72% overall retention rate
Michaud (2005)<br>NIH-AARP Diet and Health Study, [42]	567 169 adults (50-69 years) in the United States<br>Started in 1995<br>Postal questionnaires automated phone system and telephone interviews on cancer diagnoses<br><br>Evaluation Wave: Pilot of first follow-up (2002) of 11 404 eligible participants	1. Second posting of questionnaire<br><br>2. Non-responders sent postcard with toll-free number for alternative methods of data collection via automated telephone<br><br>3. Non-responders to 1 and 2 contacted for alternative method of data collection of telephone interviews by trained interviewers	59% of questionnaires were returned by mail (both mailings combined)<br><br>Less than 1% additional retained through automated phone interviews<br><br>16% additional retained from phone interviews with trained interviewers<br><br>76% overall response rate
Mills (2000), [43]	1614 grade 6 students (11-12 years), in Canada<br>Started in 1992<br>School and postal questionnaire on adolescent smoking, drinking and health<br><br>Evaluation Waves: First (1995) and second (1997) follow-ups of baseline participants<br>Postal questionnaires with reminder letters posted 2	1. Cover sheet from questionnaire with information on projected school for the following year<br><br>2. Contact with school staff and school authorities<br><br>3. Alternative methods of data collection of	70% were found through cover sheet at grade 8 (first follow-up) compared to 62% at grade 11<br><br>20% were found through the school staff or school authorities at grade 8 (first follow-up) compared to 12% at grade 11<br><br>7% of postal questionnaires were completed and found through family or contact person at grade 8 (first follow-up) compared to 16%

Novo (1999), [44]	1083 youth (16 years) in Sweden<br>Started in 1981<br>Postal and in-person questionnaires, telephone and in-person interviews on the effects of unemployment on health<br><br>Evaluation Waves: Second follow-up (1986) of baseline participants	1. In-person interviews at 5-year follow-up<br><br>2. Postal questionnaire sent to those who did not attend reunion; non-responders were sent a reminder letter<br><br>3. Telephone interviews offered to non-participants of 1 and 2<br><br>4. Personal interviews were offered to all remaining non-participants	56% of questionnaires were completed in-person<br><br>30% completed through post<br><br>7% additional retained via telephone interviews<br><br>5% additional retained via in-person interviews<br><br>98% overall retention rate
Rudy (1994), [45]	221 females (20-35 years) randomly selected from a list of runners and graduate student telephone listing, in the United States<br>Started in 1993<br>Questionnaire on the association between exercise and menstrual cycle characteristics<br><br>Evaluation Wave: Fourth follow-up (4 months) of baseline participants	1. Assigned participants to receive monetary award of $100 at end of study or 3 non-monetary gifts with study logo; 1 gift after each month of participation	Participants who received monetary incentives were significantly more likely to complete the study, 79%, than participants who received gifts, 66%<br><br>72% overall retention rate
Russell (2001)<br>Black Women's Health Study (BWHS), [46]	64 500 Black females (21-69 years) in the United States<br>Started in 1995<br>Postal questionnaires and telephone interviews on risk factors for breast cancer and risk factors associated with illness<br><br>Evaluation Wave: First follow-up (1997) of baseline participants	1. Up to 6 postings of questionnaire<br><br>2. Reminder telephone calls to non-respondents after fifth mailing<br><br>3. Alternative methods of data collection of telephone interviews offered to non-responders of 1 and 2<br><br>4. Reminder letters	55% retention rate to first mailing, retention increased by an additional 19%, 11%, 10%, 4%, and 2% for the second through sixth mailings, respectively<br><br>15% of participants who were left reminder messages returned questionnaires<br><br>Of the participants who did not respond to any of the mailings, 34% who were reached by phone agreed to an interview<br><br>Use of reminder letters or postcards resulted in about a 3% increase in retention rate<br><br>83% overall retention rate
Tolusso (2003)<br>National Population Health Survey (NPHS), [47]	17 276 people (all ages) in Canada<br>Started in 1994<br>Telephone and in-person interviews on health status and determinants of health<br><br>Evaluation Wave: Third follow-up (2003) of baseline participants	1. Attempted contact via telephone calls to participants or contact persons, directory assistance and directory searches<br><br>2. Alternative methods of data collection of personal interviews	7.2 attempted contacts were needed to complete an interview<br><br>About 90% of interviews were completed in 15 attempted contacts or less<br><br>Participants interviewed by telephone required 7.1 attempted contacts compared to 10.9 for those interviewed in person

Ullmann (1998)<br>UCLA Study of Adolescent Growth, [48]	1634 students (12-16 years) in the United States<br>Started in 1976<br>Postal questionnaires on socioeconomic status, personality and attitudinal traits, and drug use behaviour<br><br>Evaluation Wave: 16-year follow-up (1992) of 814 baseline participants<br>All participants offered $25 cheque to be sent after completion of questionnaire (not evaluated)	1. Reminder letters (up to 3)<br><br>2. Second posting of questionnaire with a monetary incentive of $25 cheque to non-responders	41% returned questionnaire without reminder, 12% returned after first reminder letter, 9% after second, 1% after final reminder and<br><br>4% returned questionnaire after receiving second questionnaire and monetary incentive<br><br>68% overall retention rate
Walker (2000)<br>British Regional Heart Study (BRHS), [49]	7735 males (aged 40-59 years) in the United Kingdom<br>Started in 1978<br>Postal questionnaires on cardiovascular disease risk factors<br><br>Evaluation Waves: First (1983-1985), second (1992) and third (1996) follow-ups of all survivors of baseline questionnaire	1. Posted 2 reminder letters	At the 5-year follow-up, 75% returned questionnaire without reminders, 14% after first reminder and 6% after second reminder<br><br>At the 14-year follow-up, 77% returned questionnaire without reminders and 13% after two reminders<br><br>At the 20-year follow-up, 71% returned questionnaire without reminders, 13% after first reminder and 5% after second<br><br>98%, 91% and 88% retention rate at 5-year, 14-year and 20-year follow-up, respectively
Women's Health Australia Research Group (2001)<br>Women's Health Australia (WHA) Study, [50-53]	14 247 women (18-23 years old), 13 716 women (45-50 years old) and 12 432 women (70-75 years old) in Australia<br>Started in 1996<br>Postal questionnaires on health and lifestyle<br><br>Evaluation Wave: Second follow-up (1998-2000) of baseline participants	1. Reminder letters<br><br>2. Reminder calls<br><br>3. Alternative methods of data collection of telephone interviews	53% of the young cohort, 86% of the middle and 74% of the older cohort returned questionnaire early (i.e. no reminder or with reminder letter)<br><br>17% of the young cohort, 1% of the middle and 6% of the older returned questionnaire after the reminder calls<br><br><1% of young cohort, 5% of middle and 8% of older cohort completed telephone surveys or interviews<br><br>71% overall retention rate among the young cohort, 92% in the middle cohort & 89% in the older cohort

[a] All money values USD, unless otherwise stated
[b] Response rates at time of publication
° Personal Communication
